# Supplementary figures and images for: Analysis of the swine movement network in Mexico: A perspective for disease prevention and control
Source: PLoS One. 2024 Aug 30;19(8):e0309369. doi: 10.1371/journal.pone.0309369 (PMC11364239; doi:10.1371/journal.pone.0309369)

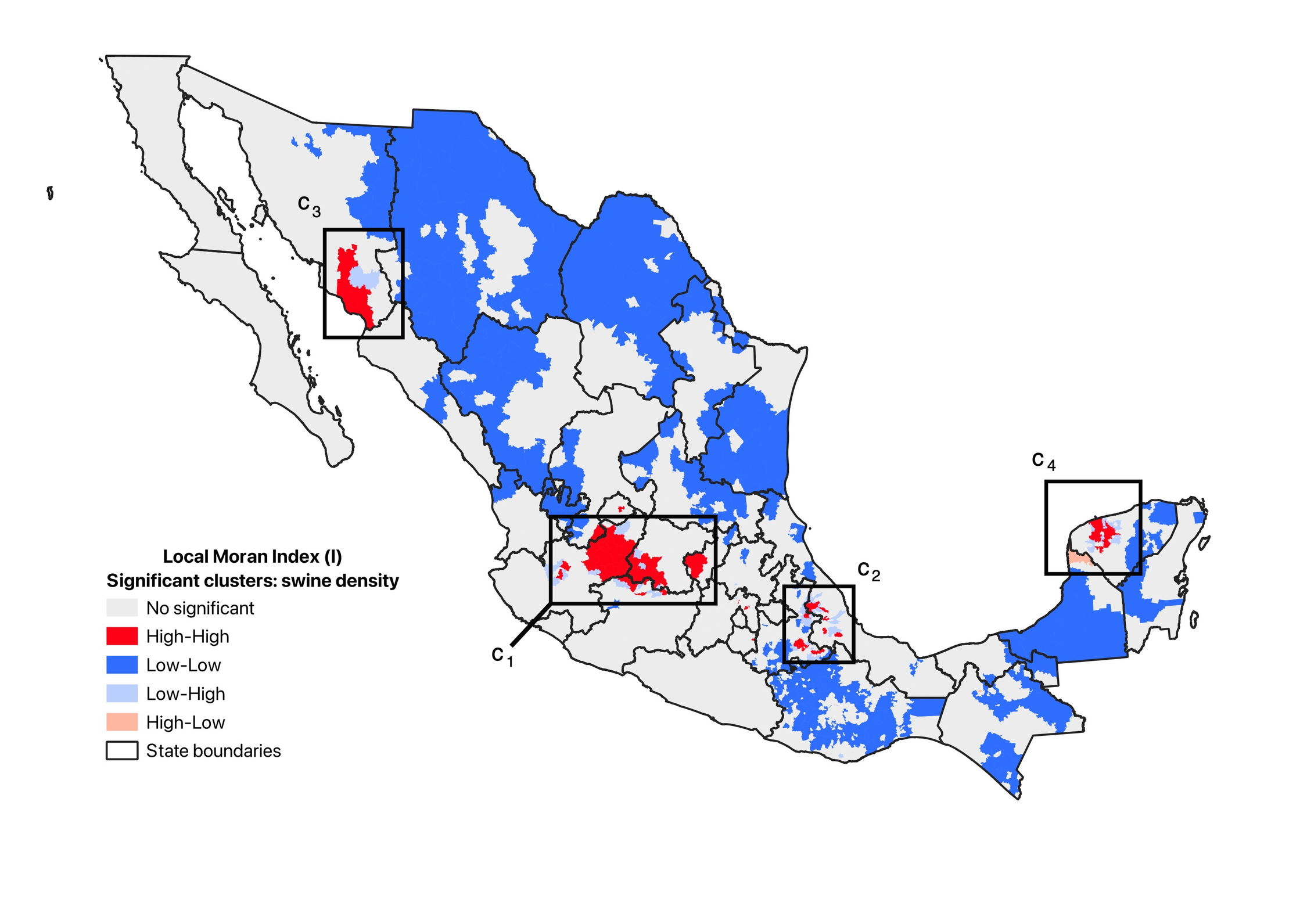

Supplement: S1 Fig — This map highlights areas in red indicating concentrated swine populations (hot spots) and regions in navy blue with lower swine density (cold spots). The map reveals areas in transition, with Low-High (light blue) and High-Low (light red) gradients. The outlined squares in the figure denote the location of swine production centers (c1, c2, c3, and c4). Note: The map of the administrative boundaries was obtained from the Marco Geoestadistico, provided by the Instituto Nacional de Estadistica y Geografia (INEGI). Source: https://www.inegi.org.mx/temas/mg/#descargas. (TIF) [file pone.0309369.s001.tif]

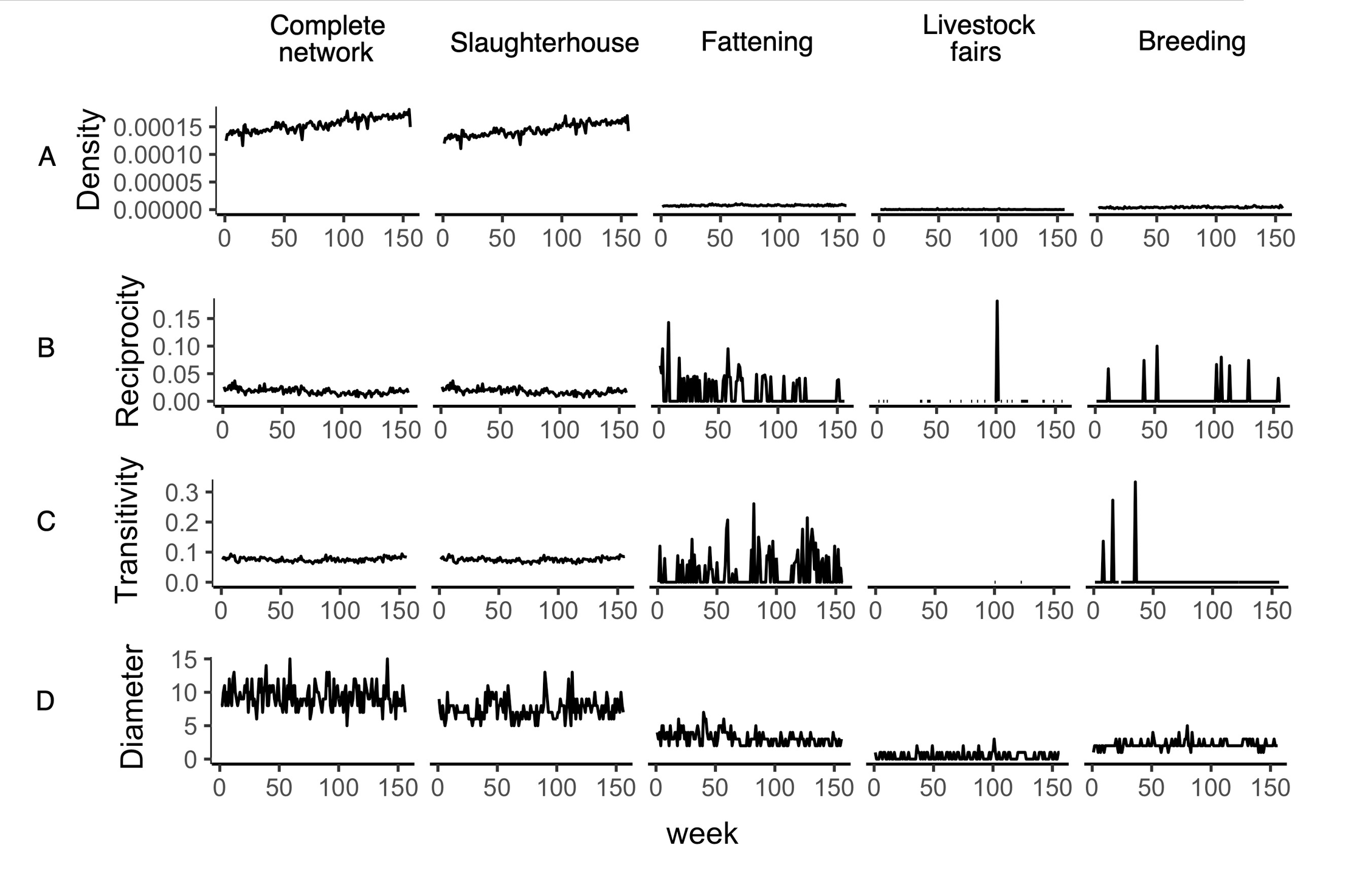

Supplement: S2 Fig — The following metrics are described: density (A), reciprocity (B), transitivity (C), and diameter (D). (TIF) [file pone.0309369.s002.tif]
