# Supplementary material for: Analysis of the swine movement network in Mexico: A perspective for disease prevention and control
Source: PLoS One. 2024 Aug 30;19(8):e0309369. doi: 10.1371/journal.pone.0309369 (PMC11364239; doi:10.1371/journal.pone.0309369)
Supplement: S1 Table — (PDF) [file pone.0309369.s003.pdf]

c_1		c_2		c_3		c_4	
**State**	**Municipality**	**State**	**Municipality**	**State**	**Municipality**	**State**	**Municipality**
**Guanajuato**	Abasolo	**Veracruz**	Carrillo Puerto	**Sonora**	Cajemé	**Yucatán**	Abala
	Manuel Doblado		Coatepec		Etchojoa		Acanceh
	Huanimaro		Cordoba		Hermosillo		Baca
	Irapuato		Perote		Huatabampo		Cacalchen
	Penjamo		Las Vigas de Ramírez		Navojoa		Conkal
	Purisima del rincón		Totutla		Benito Juárez		Chicxulub Pueblo
	Romita	**Puebla**	Ajalpan				Hoctun
	Valle de Santiago		Guadalupe Victoria				Mérida
	Yuriria		Santiago Miahuatlan				Muxupip
**Michoacán**	Cuitzeo		Tehuacán				Progreso
	Jiménez		Tepanco de López				Sacalum
	Numarán		Tlacotepec de Benito Juárez				Seye
	Penjamillo						Tahmek
	La Piedad						Tecoh
	Yurecuaro						Tixkokob
	Zinaparo						Tixpehual
	José Sixto Verduzco						Uman
**Querétaro**	Colón						
	Huimilpan						
	El Marques						
	Pedro Escobedo						
	Querétaro						
**Jalisco**	Acatic						
	Arandas						
	Atotonilco el Alto						
	Ayotlán						
	La barca						
	Cocula						
	Cuquio						
	Degollado						
	Jalostotitlan						
	Jamay						
	Jesús María						
	Mexticacan						
	Ocotlan						
	San Diego de Alejandria						
	San Juan de los Lagos						
	San Julian						
	San Miguel el Alto						
	Tala						
	Teocaltiche						
	Tepatitlan de Morelos						
	Tototlan						
	Unión de San Antonio						
	Valle de Guadalupe						
	Cañadas de Obregón						
	Yahualica de González Gallo						
	Zapotlanejo						
	San Ignacio Cerro Gordo						
