# Supplementary material for: Analysis of the swine movement network in Mexico: A perspective for disease prevention and control
Source: PLoS One. 2024 Aug 30;19(8):e0309369. doi: 10.1371/journal.pone.0309369 (PMC11364239; doi:10.1371/journal.pone.0309369)
Supplement: S2 Table — (PDF) [file pone.0309369.s004.pdf]

Metrics of nodes	Number of swine farms	Number technified swine farms	Swine density per km^2	Slaughterhouses and pork processing plants	Number of livestock fairs
Complete network					
In-degree	0.46*	0.41*	0.54*	0.39	0.36
Out-degree	0.21	0.45*	0.41*	0.31	0.12
Betweenness	0.24	0.47*	0.38	0.33	0.18
Hubs	0.41*	0.53*	0.53*	0.4*	0.29
Authorities	0.46*	0.41*	0.51*	0.38	0.35
Subnetwork for slaughterhouse					
In-degree	0.35	0.35	0.38	0.37	0.33
Out-degree	0.2	0.49*	0.41*	0.29	0.11
Betweenness	0.21	0.43*	0.36	0.32	0.16
Hubs	0.2	0.49*	0.41*	0.3	0.11
Authorities	0.35	0.38	0.43*	0.38	0.32
Subnetwork for fattening					
In-degree	0.33	0.32	0.32	0.33	0.29
Out-degree	0.18	0.4*	0.37	0.27	0.13
Betweenness	0.19	0.36	0.27	0.29	0.17
Hubs	0.24	0.42*	0.39	0.34	0.21
Authorities	0.32	0.32	0.33	0.34	0.28
Subnetwork for livestock fairs					
In-degree	0.15	0.18	0.17	0.17	0.13
Out-degree	0.11	0.21	0.17	0.17	0.1
Betweenness	0.09	0.13	0.09	0.11	0.08
Hubs	0.12	0.21	0.18	0.2	0.12
Authorities	0.16	0.21	0.19	0.19	0.15
Subnetwork for breeding					
In-degree	0.45*	0.42*	0.47*	0.34	0.33
Out-degree	0.16	0.33	0.26	0.23	0.13
Betweenness	0.17	0.31	0.24	0.25	0.16
Hubs	0.16	0.33	0.25	0.22	0.13
Authorities	0.44*	0.42*	0.47*	0.33	0.33

*Significant correlations ($P < 0.05$) with a moderate strength are marked in bold in the table
