# Supplementary material for: Analysis of the swine movement network in Mexico: A perspective for disease prevention and control
Source: PLoS One. 2024 Aug 30;19(8):e0309369. doi: 10.1371/journal.pone.0309369 (PMC11364239; doi:10.1371/journal.pone.0309369)
Supplement: S3 Table — (PDF) [file pone.0309369.s005.pdf]

Variables	Community detection algorithms	
	Random walk	Walks weighted
Total communities	11	47
Large communities*	6	6
Isolated nodes	7	440
Nodes in large communities	1305	661
Minimum nodes per community	2	2
Maximum nodes per community	637	345
Intra-community edges[% total]	6523 [78.8%]	4366 [75.7%]
Inter-communities edges	1750	1398

* Communities largest with 20 municipalities
