# Supplementary material for: Analysis of the swine movement network in Mexico: A perspective for disease prevention and control
Source: PLoS One. 2024 Aug 30;19(8):e0309369. doi: 10.1371/journal.pone.0309369 (PMC11364239; doi:10.1371/journal.pone.0309369)
Supplement: Attachment — Submitted filename: Response to Reviewers.docx [file pone.0309369.s006.docx]

**Rebuttal Letter**

**Reviewer #1**

**Line 57: Could you explain better this percentages? The pig population was counted based on the number of farms or number of pigs (could you include the numbers of the estimation), percentage of production refers also to metric tons of meat or number of slaughtered animals?**

*Thank you for your feedback. We have included the total pig population and pork production in Mexico. Please see the lines 56 – 59.*

**Line 110: Municipal is a third or fourth level political organization. Federal, State and Municipal? Could you explain this.**

*We made the changes suggested. Please see the lines 110-112.*

**Line 143: Spanish original names could be italic, or a short English name followed by the Spanish on parenthesis.**

*We used short English names followed by the Spanish names in parentheses. All changes were made in lines 24, 81, 85, 129, and 145.*

**Line 216: Please consider indicate that you used hubs and authorities score, just for general audience readers.**

*We have added an explanation about this topic and rephrased the text. Please see the lines 217 – 219.*

**Line 223: What about the semi-technified and backyard?**

*The swine population variables include data from technified, semi-technified, and backyard farms. However, we created a subset of technified farm because these types of farms frequently report to the SNAM database. Distinction between these categories of farms is explained in lines 224 - 227.*

**Line 228-241: Could you merge these two paragraphs.**

*We have rewritten the paragraph to improve readability. See lines 231-238.*

**Line: 238: Could you rephrase for better readability, why 20?**

*We observed that some communities ranged from 1 to 3 nodes, while the others had more than 27 nodes. To address this discrepancy, we established a cut-off point of 20 nodes, categorizing communities with fewer than 20 nodes as small and those with 20 or more nodes as large. Please see the explanation in the manuscript in lines 236-238.*

**Line 265: Could you include the percentage of reduction.**

*We made the adjustment in the manuscript. Please see the line 261-263.*

**Line 264: According to Fig.1C, the movement from livestock fairs and fattening reduces between Jun, Jul, Ago any explanation for this reduction (On the discussion the reduction is explained around Mar-Apr).**

*In the section of discussion, we have focused on explaining the reduction in swine movements observed during the March to April period. We attribute this decline to the influence of religious traditions, as corroborated by the available data.*

*The observed pattern of movements to fairs from June to August may be influenced by other factors, such as the scheduling of fairs or an economic factor, but we don't have the data to discuss this pattern. We made a clarification in the manuscript. Please see the lines 442 - 447*

**Line 278: According to Fig. 1C the number of shipments per month to livestock fairs, are extremely low, considering the 600.000 movements, it shows that the clients of that fairs should be backyard producers without registering those movements, is that appreciation, correct? Could you comment about that?**

*Although it is very likely that backyard producers do not participate in the registration of swine movements, we are currently unable to identify the type of farm in movement dat*a*. We compared the number of farms from the National Livestock Register (PGN) with network metrics and found a correlation specifically with technified farms. This provides indirect evidence of the influence that technified farms have on the configuration of the network. However, further research is needed in the future.*

*In the discussion we noticed these limitations. See the lines 495 – 499.*

**Line 288 Fig. 2. Could you comment about the long-distance movements (e.g. Line Merida – Chihuahua or Hemosillo >2500 km).**

*Long-distance movements are exceptional and involve a small number of animals and shipments. They are associated with the sale of animals for breeding purposes or high-value pigs. We have included an explanation of long-distance movements in the discussion to ensure the reader is fully aware of these factors. See the lines 468-470.*

**Table 2: Please consider including on Swine population the density (pigs/farms per Km2) to have a direct density comparison between hotspots, also could you consider including the local Moran index on the text or on the table for the hotspots, it could be useful (I suppose that local index should be higher than global index).**

*Thank you for the suggestion. We included the density of farms and pigs per km^2^ in Table 2. Regarding the local Moran Index, we calculate the average value per hotspot, as it is calculated individually for each municipality. The local Moran index is indeed higher than the value of the global index (I = 0.229, P < 0.05), indicating spatial clustering in the dataset.*

**Line 323: When superimposing Fig2 and SM1 seems that there are some movements close to the c3 and c4 hotspots, please consider looking further if they are related or just errors on the data as my previous comment about line 288.**

*We have reviewed the database, and we can confirm that the movements observed near the c3 and c4 hotspots are correctly recorded.* *As we mentioned in the previous commentary, these movements are exceptional with few animals and are due to shipments of high value pigs. We have included an explanation of this long-distance movement in the discussion. See the lines 468-470.*

**Line 317-323: Please consider include a percentage to the 15,148 considering the reference of total movement in the hotspots, some are explained as numbers and other as percentages, that makes harder to read.**

*We have made the suggested adjustments. See lines 345 – 349.*

**Line 333: Please consider explain what you refer when talking about similarities and discrepancies.**

*The differences and similarities were clarified in the manuscript and the paragraph was reduced for better reading on lines 327 -329.*

**Line 338: Please consider indicate the total number of farms registered on the PGN. Do you consider that they are backyard farms?**

*We have made the suggested adjustments. Please see the line 333.*

**Table 3: Euclidean distance: 95th percentile and maximum distances are quite high biological plausibility of terrestrial transport, consider checking the validity of those movements, or maybe they are aerial transport maybe for high valuable pigs.**

*We checked the database and found the records for these movements are valid. As mentioned in the previous commentaries, these movements are exceptional with very few shipments during the analysis period. The aerial transport is possible, but we cannot validate this type of information.*

*Although these movements are exceptional, we decided to keep the records in the analysis to have the highest representativeness of the movements. We also explain this long-distance movement in the manuscript. See the lines 468-470.*

***Line 439: Please consider rephrasing the loops explanation.***

*Thank you for your feedback. We have created a more concise and readable version of this paragraph. Please see the lines 415 – 419.*

**Line 366: Please consider which was the first assumption and based on what analysis.**

*We appreciate your feedback. The original text was unclear, so we have revised it to make it more concrete. Please see lines 359-364.*

**Line 404: Please consider separating methods and results in this paragraph.**

*The fragment of methods was placed in lines 217 – 219, while the results were placed in line 390-391.*

**Line 420: Please consider rephasing this paragraph.**

*The paragraph has been revised to enhance clarity and conciseness. The updated version is lines 396-400.*

**Line 425: Please consider explaining better why on the map there are NA. My understanding is that 11 communities were identified, the largest 6 are plotted on the map and the other 5 (weaker) are plotted as NA.**

*Thanks for your comment. We had an error in identifying small communities as NA on the map. We updated the map to show only the label of the large communities for improve clarity and interpretation. We also update the caption to inform readers about this issue (line 409-410).*

**Line 428: Please consider providing a clearer explanation of “geographically clearly defined” when looking at the map (Fig. 5B) as the colours appear quite scattered, particularly the blue ones. Additionally, regarding the “correspondence” of hotspots to the communities: C4 corresponds to the “red” community, but one municipality is on the opposite northeast side of the country. C2 is divided between the “blue”, “green”, “yellow”, NA (hard to tell); C1 is within the blue community, with some NA as well. Maybe a count of the match could help or use “approximate” in reference to the hotspots.**

*Although there is a geographic overlap between the hotspots and the identified communities, the correlation is not perfectly defined or clear. Therefore, we decided to omit this statement from the manuscript to avoid confusion.*

**Line 459: Please consider that this paragraph only addresses the industrial perspective, whereas backyard producers might find it easier to slaughter animals on-site. There is evidence of movements to and from markets and maintaining them for ~1o monthly movements seems economically inefficient (please consider include the number of those markets).**

*We recognize the limitations in describing the movement profile of small producers due to their lack of regularity in reporting their mobilization. The data gives us a good starting point to understand the swine movement patterns in Mexico and could inspire more focused research in the future.*

*The number of these markets has been included in the correlation analysis with the number of livestock fairs. This helped us to better understand their influence on the pig movement network.*

**Line 467: Please consider that movements to slaughterhouses are usually considered as “dead-ends” with respect to disease transmission. How the network without dead ends would look like for using in a disease transmission model?**

*A network without “dead-ends” would involve movements that eventually return to the original location or continue circulating within the network. This type of network could potentially result in increased disease transmission dynamics, as infected animals could reintroduce the pathogen back into the network instead of being removed from it through slaughter.*

*However, this is not common on farms in Mexico because animals are usually sent to slaughterhouses or points of sale after a certain fattening period. This creates a unidirectional movement that does not include a return to the farm of origin. This argument is further supported by the low percentage of reciprocity observed in the networks (Please see the Table 3).*

*We have summarized the paragraph. See the lines 477-482.*

**Line 476: Please consider rephrasing this paragraph.**

*In response to the other reviewer's request for a more concise discussion, we have decided to remove this paragraph.*

**Line 551: Please consider resume this long paragraph.**

*To be more concrete in the discussion, we have summarized the paragraph. See the lines 472 – 475.*

**Reviewer #2:**

1. **The analysis is presented at the municipality level (as nodes) and not the farm level. Hence, there are limitations in interpreting the results. These limitations should be clarified to the readers.**

*We appreciate your feedback. These limitations have already been addressed in the manuscript. It is clarified that the analysis is presented at the municipality level rather than the farm level, and the implications for interpreting the results are explained. (Please see the lines 110 - 112).*

2. **The author's estimate of about 95.9% being attributed to slaughterhouse movements could be misleading if it is just based on shipment counts rather than pig population. Heads in fattening shipments are almost four times more than slaughterhouse shipments. This should be clarified in the wording.**

*We have added the suggested clarification in the manuscript. Please see the lines 270-271.*

3. **Some of the statements in the paper are too trivial. For example, statements such as "movements occur in the same areas where swine production is concentrated..." do not provide any interesting insight. The authors should limit these statements and make the write-up concise.**

*Thank you for your feedback. We've made the manuscript more concise and removed trivial statements.*

4. **Lines 191-193: The authors use and report Euclidean distance to measure distances between municipalities/nodes. However, shipment distances computed from road networks will likely better fit this context.**

*Thank you for your feedback. However, this approach presents a significant challenge due to the lack of precise farm location data. We use the centroid of municipalities to calculate the Euclidean distance. Consequently, using road networks would not improve the accuracy of our distance estimates. Given the area differences between municipalities, it would be difficult to accurately estimate the most appropriate road routes. This could lead to confusion and redundancy, as all trips would originate from the same node and geographic location.*

5. **The discussion section is too long, where the authors provide a lot of commentary, but for the reader, it is difficult to extract information that could be used in later studies. For example, what are the top 2 or 3 insights/findings that are novel contributions of this paper?**

*We agree with your opinion, and we have adjusted the discussion to make it more concise.*

*In response, we have identified 3 contributions of this paper:*

1. *This study describes for the first-time swine movement patterns in Mexico, providing valuable information that can strengthen disease control and prevention strategies and improve the effectiveness of swine traceability procedures.*
2. *Identification of significant nodes and commercial communities: The analysis identified four main hotspots that concentrate the largest pig population in Mexico, revealing a complex flow of pig movements that also have connections with the rest of the municipalities in the country.*
3. *Our results mainly reflect the large-scale swine industry in Mexico, but more research is needed to understand the dynamics of smallholder swine movements.*
1. **Lines 262-264: Grammar issues.**

*We have considered your recommendation and have made the text more concise. Please see lines 259-260.*
